# How to Manage COVID-19 Vaccination in Immune-Mediated Inflammatory Diseases: An Expert Opinion by IMIDs Study Group

**DOI:** 10.3389/fimmu.2021.656362

**Published:** 2021-04-15

**Authors:** Francesca Ferretti, Rosanna Cannatelli, Maurizio Benucci, Stefania Carmagnola, Emilio Clementi, Piergiorgio Danelli, Dario Dilillo, Paolo Fiorina, Massimo Galli, Maurizio Gallieni, Giovanni Genovese, Valeria Giorgi, Alessandro Invernizzi, Giovanni Maconi, Jeanette A. Maier, Angelo V. Marzano, Paola S. Morpurgo, Manuela Nebuloni, Dejan Radovanovic, Agostino Riva, Giuliano Rizzardini, Gianmarco Sabiu, Pierachille Santus, Giovanni Staurenghi, Gianvincenzo Zuccotti, Pier Carlo Sarzi-Puttini, Sandro Ardizzone

**Affiliations:** ^1^ Gastroenterology Unit, ASST Fatebenefratelli-Sacco, Department of Biomedical and Clinical Sciences (DIBIC) L. Sacco, Università degli Studi di Milano, Milan, Italy; ^2^ Rheumatology Unit, S. Giovanni di Dio Hospital, Azienda USL-Toscana Centro, Florence, Italy; ^3^ Unit of Clinical Pharmacology, Department of Biomedical and Clinical Sciences (DIBIC) L. Sacco, Università degli Studi di Milano, Milan, Italy; ^4^ Scientific Institute IRCCS E. Medea, Lecco, Italy; ^5^ Surgery Unit, ASST Fatebenefratelli Sacco, Department of Biomedical and Clinical Sciences (DIBIC) L. Sacco, Università degli Studi di Milano, Milan, Italy; ^6^ Pediatric Department, Ospedale dei Bambini, ASST Fatebenefratelli Sacco, Department of Biomedical and Clinical Sciences (DIBIC) L. Sacco, Università degli Studi di Milano, Milan, Italy; ^7^ Division of Endocrinology, ASST Fatebenefratelli - Sacco, Milan, Italy; ^8^ International Center for T1D, Pediatric Clinical Research Center Romeo ed Enrica Invernizzi, DIBIC, Università Degli Studi di Milano, Milan, Italy; ^9^ Nephrology Division, Boston Children's Hospital, Harvard Medical School, Boston, MA, United States; ^10^ Department of Biomedical and Clinical Sciences (DIBIC) L. Sacco, Università degli Studi di Milano, III Infectious Diseases unit, University Hospital “Luigi Sacco”, Milan, Italy; ^11^ Department of Biomedical and Clinical Sciences (DIBIC) L. Sacco, Università degli Studi di Milano, Milan, Italy; ^12^ Nephrology and Dialysis Unit, “L. Sacco” Hospital, ASST Fatebenefratelli-Sacco, Milano, Italy; ^13^ Department of Pathophysiology and Transplantation, Università degli Studi di Milano, Milan, Italy; ^14^ Dermatology Unit, Fondazione IRCCS Ca' Granda Ospedale Maggiore Policlinico, Università degli Studi di Milano, Milan, Italy; ^15^ Rheumatology Unit, Department of Biomedical and Clinical Sciences (DIBIC) L. Sacco, Università degli Studi di Milano, Milan, Italy; ^16^ Eye Clinic, Department of Biomedical and Clinical Sciences Luigi Sacco, Università degli Studi di Milano, Milan, Italy; ^17^ The University of Sydney, Save Sight Institute, Discipline of Ophthalmology, Sydney Medical School, Sydney, NSW, Australia; ^18^ Pathology Unit, Department of Biomedical and Clinical Sciences (DIBIC) L. Sacco, Università degli Studi di Milano, Milan, Italy; ^19^ Division of Respiratory Diseases, Ospedale L. Sacco, ASST Fatebenefratelli-Sacco, Milan, Italy; ^20^ Department of Infectious Diseases, ASST Fatebenefratelli-Sacco, Università degli Studi di Milano, Milan, Italy; ^21^ School of Clinical Medicine, Faculty of Health Science, University of the Witwatersrand, Johannesburg, South Africa

**Keywords:** COVID-19, Sars-CoV-2, vaccine, IMIDs, chronic disease, prevention

## Abstract

Since March 2020, the outbreak of Sars-CoV-2 pandemic has changed medical practice and daily routine around the world. Huge efforts from pharmacological industries have led to the development of COVID-19 vaccines. In particular two mRNA vaccines, namely the BNT162b2 (Pfizer-BioNTech) and the mRNA-1273 (Moderna), and a viral-vectored vaccine, i.e. ChAdOx1 nCoV-19 (AstraZeneca), have recently been approved in Europe. Clinical trials on these vaccines have been published on the general population showing a high efficacy with minor adverse events. However, specific data about the efficacy and safety of these vaccines in patients with immune-mediated inflammatory diseases (IMIDs) are still lacking. Moreover, the limited availability of these vaccines requires prioritizing some vulnerable categories of patients compared to others. In this position paper, we propose the point of view about the management of COVID-19 vaccination from Italian experts on IMIDs and the identification of high-risk groups according to the different diseases and their chronic therapy.

## Highlights

- Vaccination is the mainstay for the prevention of COVID-19 diffusion. To date, two mRNA COVID-19 vaccines and one viral-vectored vaccine received EMA authorization with a satisfactory efficacy and a good safety profile.
- Although real data on efficacy and safety of COVID-19 vaccines in specific subset of patients still have to be defined, patients with IMIDs can reasonably be encouraged to get vaccinated in most cases, according to previous available experiences and experts’ opinion.
- A priority grading should take into account demographic and geographical differences, different occupational exposures and IMIDs-related risk factors, including any factors that lead to the exclusion or delay of vaccine administration (e.g. patients with history of severe allergy, ongoing SARS-CoV-2 infection, patients on steroid or rituximab therapy).

## Introduction

In December 2020, in the United States FDA approved with an Emergency Use Authorization (EUA) the COVID-19 vaccine Pfizer-BioNTech (BNT162b2) for the prevention of Sars-CoV-2 infections in individuals aged 16 years and older (1), and Moderna (mRNA-1273) (2) in subjects aged 18 years and older. In January 2021, EMA authorized the COVID-19 vaccine AstraZeneca (ChAdOx1 nCoV-19) in people aged 18 and older (3).

Children and adolescents outside of these authorized age groups should not receive COVID-19 vaccination at this time as well as pregnant women, since they have been excluded from the trials (4, 5).

The BNT162b2 and mRNA-1273 vaccines are chemically modified mRNA vaccines expressing the prefusion spike glycoprotein of Sars-CoV2 packaged in lipid nanoparticles for effective cellular delivery. The early encouraging results were reported in September 2020, demonstrating that the mRNA-1273 vaccine results in high amounts of binding and neutralizing antibodies in young and old adults (6). In December, this vaccine was shown to possess a 94.1% efficacy for the prevention of symptomatic COVID-19 (5). In parallel, the BNT162b2 vaccine demonstrated the 94.6% efficacy after two doses spaced 21 days apart (4). The ChAdOx1 nCoV-19 consists of a replication-deficient adenoviral vector expressing the full-length Sars-CoV2 spike protein (7). This viral-vectored vaccine exploits the ability of adenovirus to infect human cells thus delivering double stranded DNA coding for the spike protein of Sars-CoV2. The ChAdOx1 nCoV-19 vaccine shows a 82.4% efficacy after two doses given 12 weeks apart (7).

These vaccines raise no severe safety concerns except for temporary local reactions, such as injection-site events (pain, erythema, induration, and tenderness), and minor systemic adverse events, i.e. fatigue and headache (4, 5, 7).

According to the fact sheet for Pfizer-BioNTech and Moderna vaccine providers, the main contraindication to the administration of the vaccine is a known history of a severe allergic reaction to any component of the vaccines. To date, no other absolute contraindication has been reported (2, 8).

Due to reports of anaphylactic reactions in Pfizer-BioNTech COVID-19 recipients, an interim clinical consideration from the Advisory Committee on Immunization Practices (ACIP) of the Center of Disease Control and Prevention (CDC) suggests to investigate and consider a history of severe allergic reaction also to any other vaccine or injectable therapy as a precaution but not a contraindication in both vaccines. Of course, it is recommended by FDA to have adequate medical equipment immediately available to manage a potential allergic reaction after the administration (8, 9).

As regards immunocompromised or autoimmune patients, no data are currently available on safety and efficacy of mRNA COVID-19 vaccines. HIV patients with stable infection were included in trials (196 patients), but no data are published on this cohort of patients so far (4). However, no specific contraindications to vaccination have been reported. Notably, due to the unknown safety profile and effectiveness as well as the potential reduced response in an immunocompromised system, it is recommended to continue adopting adequate measures of protections (10).

Among the warnings, it is reported that Pfizer-BioNTech Vaccine could not be effective in all the recipients. In particular, immunocompromised persons, including patients on immunosuppressant drugs, could have a lower immune response to Pfizer-BioNTech vaccine. However, patients under immunosuppressive therapy or immunocompromising conditions have been excluded from the trial (4).

Between the end of December 2020 and the beginning of January 2021, EMA’s Committee for Medicinal Products for Human Use (CMPH) authorized the marketing applications for Pfizer-BioNTech (11) and Moderna (12) COVID-19 vaccines in an expedited manner, evaluating the data on their quality, safety and efficacy (11). On January 29^th^ 2021, EMA’s CMPH approved the AstraZeneca COVID-19 vaccine (3). Therefore, all authorized COVID-19 vaccines in Europe and U.S. are non-live-vaccines (viral mRNA vaccines and nonreplicating viral vector vaccines); nevertheless, to date, more vaccines with different mechanisms of action (non-replicating viral vector vaccines, protein subunit, inactivated virus-based vaccines) are under evaluation in Phase 2 and Phase 3 clinical trials.

The availability of the vaccines poses the question whether potential vulnerable categories of patients, including patients with immune-mediated inflammatory diseases (IMIDs) such as inflammatory bowel disease (IBD), rheumatologic, cutaneous, kidney, ocular, pulmonary chronic diseases and diabetes would be candidates and their priority to undergo vaccinations. The role of COVID-19 vaccine in these categories has not been explored yet by population-based studies as these subjects have been excluded by the available trials. Due to the lack of data in literature, in this opinion paper we report different experts’ opinions for each disease and a paragraph from infectiologists about the COVID-19 vaccination in patients with immune mediated chronic diseases. Furthermore, we discuss about the scheduled time of the vaccination in patients under immunosuppressive or biological therapy (**Table 1**).

**Table 1:** Interaction between medications and the timing of COVID-19 vaccination.

Medications	Timing of vaccination
**No interaction**		*Mesalamine, sulfasalazine* *Azathioprine* *Mycophenolate* *Anti-TNFα* *Anti-ILs (-1, -4, -5, -5R, -6R, -12/23, -13, -17, -23)* *Anti-integrins* *Leflunomide* *Belimumab* *Oral calcineurin inhibitors* *Cyclophosphamide (oral)* *Hydroxychloroquine* *Low-dose corticosteroids (prednisone-equivalent dose <20 mg/day)* *Intravenous immunoglobulin*	No modification needed
**With interaction**	
*High-dose corticosteroids (prednisone-equivalent dose >20 mg/day)*	If a corticosteroids tapering is not possible, evaluate case by case
*Methotrexate*	Hold one week after each vaccine dose*
*JAK inhibitors*	Hold one week after each vaccine dose*
*Abatacept SC/IV*	SC: for the first dose of the vaccine, hold one week prior to and one week after IV: administer the first dose of the vaccine four weeks after the Abatacept infusion and the following Abatacept infusion with a 1 week delay
*Cyclophosphamide IV*	One week delay after the vaccine administration*
*Rituximab*	Patients who have not started rituximab need to be vaccinated ≥ 4 weeks prior to rituximab infusion, while patients under rituximab need to be vaccinated 12-20 weeks after completion of a treatment cycle

TNFα, tumor necrosis factor; IL, interleukin; IV, intravenous; SC, subcutaneous.

*in well-controlled disease. Adapted from the American College of Rheumatology Guidelines (13), the British Society of Gastroenterology Position statements (14) and others (15, 16).

## Experts’ Opinion

### Inflammatory Bowel Diseases

It is known that IBD patients present no alteration of immunocompetence due to the disease itself (17), even though they might be at higher risk of infections in relation to the concomitant immunosuppressive therapy, age, co-morbidities, malnutrition, parenteral nutrition, and bowel surgery (18). Among available therapies, the main drugs increasing the risk of infection are corticosteroids (a total daily dose equivalent to ≥20 mg of prednisolone for ≥2 weeks). However, all immunomodulators including thiopurines, methotrexate, calcineurin inhibitors, and biologics, affect the immune response of patients, especially if used in combination therapies (19, 20). This is the reason why before starting the therapy, IBD patients should undergo screening tests for main infectious diseases and/or vaccination for hepatitis B and varicella zoster virus. Moreover, an annual influenza vaccination is suggested for patients on immunosuppressant or biological therapy, due to the higher risk of developing a severe or complicated form of the infection (19).

With the outbreak of the Sars-CoV-2 pandemic, vaccination against influenza became further recommended in the whole population, due to the difficulty in discriminating between COVID-19 and other respiratory infections. Analogously, also pneumococcal vaccination is recommended in patients with IBD, especially if immunosuppressed and if aged 60 and over (21).

In the last year, several studies were conducted to evaluate a potentially higher susceptibility of IBD patients to COVID-19 due to concomitant chronic inflammation and immunosuppressive treatment. However, this hypothesis was not confirmed by clinical studies (22). In particular, one Italian study concluded that immunosuppressive therapy was not significantly associated with severe COVID-19, even though active IBD in the elderly with comorbidities were predictive factors of severe infection (23).

All the available studies agree that the biological therapies with a systemic action are not a risk factor for higher incidence of COVID-19 or a more aggressive disease. Actually, anti-TNFα drugs and Ustekinumab could have a potential protective effect due to the down-regulation of cytokine storm in course of COVID-19 (24). Conversely, patients treated with vedolizumab (anti-integrin α4β7) did not benefit from this effect, probably due to the gut-selective action of the drug (24). Notably, patients on combination therapy and thiopurines seem to be at higher risk of severe COVID-19 (25).

The availability of COVID-19 vaccines raises the question if IBD patients, especially if treated with immunosuppressant or biological therapy, should be vaccinated and which priority scale should be adopted.

To date, despite the lack of evidence about the safety and efficacy of COVID-19 vaccines on IBD patients as this cohort has been excluded from available trials, it is still possible to draw some general indications based on the available studies and previous experiences.

It is likely that non-immunosuppressed inactive IBD patients such as untreated or mesalamine-treated patients could be compared to the general population and likewise, with the same priorities, undergo the vaccination, if no absolute contraindication to the vaccine is reported. In case of ongoing immunosuppressant or biological therapy, no actual evidence contraindicates or strongly recommends and prioritizes the vaccine administration, especially since these patients already undergo the annual influenza vaccine. However, it is known that up to 20% of IBD patients treated with infliximab develop an allergic reaction to the infusion mainly due to the murine component of the drug (26). Thus, we could probably suggest caution in this subgroup of patients, due to the warning of FDA about the relative contraindication in patients with severe allergic reaction to previous infusions (8), considering also the possible reported protective effect of anti-TNFα and Ustekinumab.

The efficacy of COVID-19 vaccine in IBD patients, especially if treated with immunomodulatory therapies, should be controlled and monitored. According to available studies on other vaccines, a blunted response or a rapid waning of antibody titers could occur (27–29). However, up to now there is no reason to exclude treated IBD patients from COVID-19 vaccination, considering that an incomplete protection is better than none (30). Instead, to increase the rate of immune response, a proper time of vaccination can be suggested according the ongoing treatment (**Table 1**) and/or a different vaccination protocol with repeated booster can be evaluated.

Accordingly, the recent position statement from the British Society of Gastroenterology and IBD Clinical Research Group strongly recommends the vaccination in IBD patients, even if some concerns on the effectiveness of the immune response are raised (14).

Moreover, in a hypothetical scale of priorities, different factors should be taken into account, including i) common risk factors unrelated to IBD such as age > 65 years, cardiovascular comorbidities, and obesity; ii) IBD-related risk factors, like steroid use, active disease); iii) regional prevalence of COVID-19 and occupational exposure to the infections, i.e. health care workers, teachers, etc. (31). Of course, when different vaccines will be available, also the mechanism of action could influence the decision (32).

On this basis, we recommend that IBD patients undergo COVID-19 vaccination but continue to utilize preventive measures such as hand-washing, wearing masks and social distancing.

### Rheumatic Diseases

Patients with rheumatic diseases (RMD) may be at higher risk of infections, due to the disease activity and immunosuppressive treatment (33). Disease activity, co-morbidities, immunosuppressive drugs including glucocorticoids (GCs) and disease-modifying antirheumatic drugs (DMARDs) are all considered risk factors for infective complications. However, to date there are conflicting results about the incidence of SARS-CoV-2 infection in patients with RMD (34). Numerous data have shown an increased incidence in patients with RMD (35), in particular with a possible association with prednisone dose > 10 mg/day, while biological and targeted synthetic DMARD therapy seem to be indifferent (36, 37).

Data about SARS-CoV-2 vaccination in patients with RMD are still lacking. Patients with immunodeficiency and autoimmune diseases were included in the trial with BNT162b2 but the data have not been published yet (4, 34). The potential interference of RMD therapies in vaccine immunogenicity is an open question. Studies suggest that influenza and pneumococcal vaccines are well tolerated and generally immunogenic during DMARD use (38–42), although DMARDs may limit humoral responses to vaccines (38, 39, 43–48). Anyway, the proportion of patients reaching protective titers is generally similar in those taking DMARDs compared with control rheumatoid arthritis patients (33, 49). Therefore, limited humoral response should not preclude immunization against vaccine-preventable disease. An important exception are anti-CD20 antibodies (such as rituximab), which could inhibit protective immunity following infection and vaccination (50). Protective neutralizing antibodies and vaccination responses are expected to be attenuated until naive B cell repopulate (51).

In conclusion, there is no univocal evidence about a higher risk of contracting COVID-19 among RMD patients, and there are still no data about SARS-CoV-2 vaccination in an immunosuppressed RMD population. Notwithstanding, given the particular fragility and the susceptibility to infections of this population and the acceptable immunogenic levels reached even with DMARD therapies, immunization against vaccine-preventable diseases is advised. For anti-CD20 treatment, it is suggested that dose interruption may be undertaken to maintain control of the inflammatory disease while allowing an effective immunization against SARS-CoV-2 (**Table 1**).

### Immune-Mediated Glomerular Diseases

An altered immune reaction to self-antigens and infections represents the leading cause of immune-mediated kidney diseases, including glomerular disease (GD) and interstitial nephritis. The immune response to these antigens damages glomerular structures through immune complex formation in the glomeruli, complement activation, and injury-induced by circulating inflammatory and resident glomerular effector cells (52).

Most GD patients should have a normal immune response after vaccination, but GD treatments, including immunosuppressants, cytotoxic agents, and biological therapy, may increase the infection risk. Furthermore, patients with nephrotic syndrome (NS) are more prone to develop infections due to immunoglobulin loss in the urine (53). For these reasons, all immune-mediated GD patients should undergo vaccination for hepatitis B and varicella-zoster virus before starting the therapy with corticosteroids, cyclophosphamide, cyclosporine, tacrolimus, mycophenolic acid, and rituximab (anti-CD20).

The Kidney Disease Improving Global Outcomes (KDIGO) 2020 clinical practice guideline on GD (https://kdigo.org/guidelines/gn/) recommends pneumococcal vaccination and the annual influenza vaccination for adults and children with GD and NS. Vaccination response does not seem to be impaired by concurrent corticosteroid therapy in this cluster of patients. KDIGO also suggests both a meningococcal conjugate vaccine (MenACWY) and a serogroup B meningococcal vaccine (MenB) for patients receiving complement antagonists, such as Eculizumab (anti-c5).

However, vaccination with live vaccines (measles, mumps, rubella, varicella, rotavirus, yellow fever) is contraindicated while on immunosuppressive or cytotoxic agents. It should be deferred until immunosuppressive agents have been stopped for at least one to three months. In patients treated with steroids, prednisone dose should be less than 20 mg/day to administer live vaccines (54).

Although immunodeficiency may impair the response to vaccines in patients with IMIDs affected by chronic kidney disease (CKD), including those receiving dialysis or previously transplanted (55), vaccination remains a crucial preventive measure due to its favorable safety profile and the high rate of several infections, including Sars-CoV2, in renal patients (56). Advanced CKD appears to be associated with an increased risk of severe infection, although causality remains unclear (57, 58). Moreover, results from the International Registry of COVID infection in glomerulonephritis confirmed higher mortality (15% vs. 5%) and an increased risk of acute kidney injury (AKI) (39% vs. 14%) in this population compared with controls (59).

Interestingly, however, immunosuppressive treatment before the onset of SARS-CoV2 infection was not associated with increased mortality or AKI in GD patients, not supporting the discontinuation of these medications. On the other hand, impaired renal function itself is the main risk factors for infections (57).

Given the outcomes observed by Waldman et al. (59), we should define the timing and priority of COVID-19 vaccination in patients with IMIDs associated with CKD, especially when pharmacologically immunosuppressed, considering how effective and safe the vaccine will be. Based on the available evidence, we believe that GD patients, more prone to develop severe clinical manifestations of SARS-CoV-19 infection, should undergo the COVID-19 vaccination because of a favorable risk/benefit ratio. It is advisable to monitor the vaccine response in immune-mediated GD patients, especially if they are also affected by CKD, to understand if a different vaccination schedule is advisable based on the immunological response.

Concerning the effectiveness of the vaccine, patients on therapy with rituximab (anti-CD20) deserve special consideration (60). There is a relatively poor vaccine response in anti-CD20 treated patients due to humoral immunity inhibition through B-lymphocyte depletion, and this could affect the response to COVID-19 vaccine (51). Timing of immunosuppressive treatment is relevant. Cho et al. demonstrated that extending interval dosing or interrupting dosing to allow immature B cells to recover could grant good responses to vaccines while maintaining low levels of pathogenic memory B cells (61).

By monitoring CD-19 levels, anti-CD-20 treatment could be withheld in asymptomatic patients, and the COVID-19 vaccine prescribed with the best timing. In patients with active disease who need to continue the treatment, the vaccine could be administered towards the end of the treatment cycle, before the next dose is due. Immunosuppression could be resumed two to four weeks after the vaccination (**Table 1**). Through this approach, we would maintain the autoimmune disease under control while allowing effective immunity against SARS-CoV-2.

### Cutaneous Diseases

The outbreak of COVID-19 deeply affected the management of immune-mediated chronic-relapsing skin disorders, such as psoriasis, atopic dermatitis and hidradenitis suppurativa, as well as autoimmune bullous diseases. Patients with the above-mentioned disorders need to be adequately informed on the benefit-risk balance of COVID-19 vaccination, principally because they are frequently under conventional immunosuppressive treatments or biologic agents (62).

In a recent South-Korean cross-sectional study using nationwide claim data, alopecia areata, atopic dermatitis, psoriasis, rosacea and vitiligo did not appear to increase the susceptibility to COVID-19 or the mortality from COVID-19 (63). Similar conclusions were achieved by the European Task Force for Atopic Dermatitis (64).

Nevertheless, other authors hypothesized that systemic comorbidities related to immune-mediated chronic skin diseases, such as cardiovascular diseases, diabetes and obesity, might increase the risk of severe COVID-19 or hospitalization (65). For this reason, COVID-19 vaccination in these patients should be recommended. In our opinion, COVID-19 vaccination should be encouraged also in patients under conventional immunosuppressants or biologics, although the risk of reduced immunogenicity of COVID-19 vaccine cannot be ruled out.

Veenstra et al. found that immunosuppressants for immune-mediated cutaneous disorders were not associated with a significantly higher risk of COVID-19 or severe sequelae (66). Moreover, Cho et al. concluded in their epidemiological study that biologics for dermatological conditions did not seem to influence the susceptibility or mortality related to COVID-19, suggesting that biologics might be continuously used during the COVID-19 pandemic (63). As proof of this, Gisondi et al. found no negative impact of biologics on COVID-19 outcome in patients with psoriasis and did not advise discontinuation of biologic treatment of psoriasis to reduce the risk of SARS-CoV-2 infection (67). Data on a small cohort of patients with moderate-to-severe atopic dermatitis treated with dupilumab, a monoclonal antibody inhibiting the shared receptor component for IL-4 and IL-13, also suggest no increased risk for COVID-19 in this subset (55). Similar conclusions were drawn for patients with hidradenitis suppurativa treated with the anti-tumor necrosis factor (TNF) α adalimumab, who showed a low prevalence of COVID-19 (1%) in a high-epidemic area (Milan, Italy) (68).

However, provided that any therapeutic decision should be based on a case-by-case approach, most Dermatology Societies and expert panels recommend patients to withdraw or delay immunosuppressant drugs or biologics in case of COVID-19 diagnosis up to infection recovery, except for systemic corticosteroids, whose tapering to ≤10 mg/day of Prednisone or equivalent should be considered (62, 69–73).

On the other hand, regardless of immunosuppressive/biologic treatment, annual influenza vaccination (except live intranasal influenza vaccines) is recommended for all patients with immune-mediated skin diseases and autoimmune bullous diseases and pneumococcal vaccination is advised in specific high-risk subgroups of the same population (62, 69, 72).

The concern of potentially reduced immunogenicity of COVID-19 vaccine is well-founded, especially for autoimmune bullous disease patients who had been treated with the anti-CD20 monoclonal antibody rituximab (which temporarily depletes B lymphocytes) up to 6-12 months before vaccination (60, 74). However, currently there is insufficient evidence to ascertain whether COVID-19 vaccine effectiveness is diminished also by higher doses of conventional immunosuppressants or other biological agents such as anti-TNFα.

We recommend to timely schedule COVID-19 vaccination in autoimmune bullous disease patients treated with rituximab. Similarly to influenza vaccine recommendations, patients who have not started rituximab need to be vaccinated ≥ 4 weeks prior to rituximab infusion, while patients under rituximab need to be vaccinated 12-20 weeks after completion of a treatment cycle (**Table 1**) (15).

It ought to be noted that exacerbation of immune-mediated skin disorders following vaccination is a possible, albeit rare, event (75, 76), which, however, must not be considered as a contraindication to COVID-19 vaccination. Finally, given that COVID-19 associated mucocutaneous manifestations have been widely reported (77, 78), vaccine-related skin reactions could occur, which, however, must not be considered an argument against mass vaccination program.

### Ocular Diseases

Uveitis are a broad family of potentially sight-threatening diseases characterized by the inflammation of the vascular tunic of the eye that can be of infectious or autoimmune etiology. While infectious uveitis usually resolve after an appropriate antibiotic/antiviral/antifungal therapy, autoimmune uveitis need long term anti-inflammatory/immunosuppressive treatment often including high dose systemic steroids, steroid sparing agents and biologics (79). Uveitis patients undergoing such treatment share the same risks as all the other subjects suffering from other IMIDs and treated with a similar approach.

Since the beginning of the pandemic, many reports have focused on the ocular involvement in COVID-19. Apparently, SARS-CoV-2 can be found at several levels in the ocular tissues, from the conjunctiva (80) to the retina (81) even though no specific uveitis have been associated to COVID-19. By contrast, several authors have highlighted the presence of non-specific signs of retinal perfusion impairment (82, 83) as well as the occurrence of sight threatening thrombotic events affecting the retinal vasculature in COVID-19 patients (84, 85).

Finally, many patients with chronic ocular diseases like autoimmune uveitis and retinal diseases need frequent access to health care facilities to have their conditions monitored and treated (86). During the pandemic, many of these subjects have skipped their appointments due to rescheduling, local transportation difficulties or simply scared by the possibility of getting infected (86). Preliminary reports have already shown the dramatic consequences of treatment delays, with many patients becoming legally blind (87, 88).

The eye is an immune sanctuary and for this reason it is one of the most targeted body districts in autoimmune diseases (89). As such any external stimulus, including antigens contained into vaccines, could act as triggers and unleash an autoimmune response against ocular tissues, causing uveitis. Despite this, in the last 20 years only 300 cases of vaccine-associated uveitis have been reported in literature (90). Considering the extremely high number of vaccinations performed in this length of time, vaccine-related uveitis can be reasonably considered a very uncommon complication. There are not enough data so far to predict whether COVID-19 vaccination will induce a rate of vaccine-associated uveitis higher than other vaccines, but the results from the clinical trials have not highlighted such increased risk (4).

To conclude, most patients with autoimmune uveitis are treated with systemic immunosuppressive drugs and need frequent access to health care facilities for the accurate monitoring of their ocular conditions. In addition, COVID-19 can have negative effects on the retinal vasculature while the ocular side effects of the vaccine are very unlikely to happen. For this reason, it seems reasonable to recommend vaccination to patients with autoimmune ocular diseases unless contraindicated by specific systemic conditions they could suffer from.

### Diabetes Mellitus

Diabetes is a risk factor for severe COVID-19 and is associated with poorer outcomes (91).

Compared to age-matched controls, adult patients with T1D have a higher risk of viral, bacterial, and fungal infections, infection-related hospitalization and death (92). The risk is correlated with poor glycemic control (93). Altered immune functions, as of chemotaxis, phagocytosis and cytokines secretion, are among the proposed mechanisms (94). Individuals with diabetes should be vaccinated as follows: (i) routinely recommended immunization for children as indicated by age; (ii) annual immunization against influenza for all people above 6 months of age; (iii) immunization against pneumococcal disease, (iv) immunization for hepatitis B in not-immunized adults with diabetes aged 18 through 59 years. Moreover, immunization should be considered for not-immunized adults with T1D aged ≥60 years (95, 96). Although an impaired immune response to vaccination was postulated in patients with diabetes, no evidence has been provided (97–99).

Evidence suggests that patients with diabetes, particularly the elderly with type 2 diabetes (T2D), are at a higher risk of severe disease or death due to Sars-CoV-2 infection than individuals without diabetes (100). The underlying mechanism is not completely understood, with stronger inflammatory response and vascular dysfunction being the major supposed players. Data on children, adolescents and young adults with T1D and positive for COVID-19 have been published. All of them were asymptomatic or had a mild course of the disease (101), consistently with age-matched controls without diabetes. In a different way, new evidences show that adult with T1D are at higher risk for in-hospital death when experiencing COVID-19 after adjustment for age, sex, socio-economic deprivation, ethnicity, and geographical region (102). Interestingly, the risk has been correlated to cardiovascular and renal complications, but also to glycemic control and body mass index.

T2D increases the mortality risk in patients with COVID-19, particularly in those with more severe disease (103). The presence of poor glucometabolic control is associated with a further increase of mortality as compared to those patients within target (103). An HbA_1c_ value above the median of 7.5% was associated with worst outcomes, and only a BMI higher than 29 kg/m^2^ appeared to be strongly associated with mortality. Particularly, the presence of cardiorenal or cerebrovascular complications worsen drastically the mortality. Given that adult T1D and T2D patients are a more vulnerable population for severe forms of COVID-19, given the absence of contraindications for COVID-19 immunization, patients with T1D and T2D should represent a priority group to receive COVID-19 vaccine.

### Pulmonary Diseases

Immune and non-immune-mediated chronic lung diseases increase the risk of severe outcomes of COVID-19. Here we summarize current evidence on different lung diseases.

COVID-19 may precipitate and rapidly worsen interstitial lung diseases (ILDs), a heterogeneous group of pulmonary disorders including idiopathic pulmonary fibrosis (IPF) (104, 105). Patients with ILDs are often exposed to prolonged high dose systemic corticosteroids, immunosuppressant therapy or antifibrotics (in case of IPF). A recent European multicenter cohort study showed a higher risk of death in patients with ILDs hospitalized with COVID-19 compared with age, sex and comorbidity matched controls (HR 1.60;95%CI:1.17-2.18; p=0.003), which was further increased in patients with IPF (HR 1.74; 95%CI:1.16-2.60; p=0.007) (106, 107). All patients affected by ILDs, and especially those with IPF, should necessarily be given a prioritized access to COVID-19 vaccination programs (107). Currently, there is no evidence of a negative interaction between antifibrotics used in IPF such as nintedanib and pirfenidone and mRNA COVID-19 vaccines.

As regards chronic obstructive pulmonary disease (COPD), the available evidence does not support the hypothesis that these patients are at heightened risk of SARS-CoV-2 infection compared with the general population. However, COPD represents an independent risk factor for hospitalization (108) and it is associated with a more severe disease and unfavorable outcomes in COVID-19 patients (109). In general, COPD patients are at elevated risk of infectious events that can lead to severe exacerbations of the disease, hospitalization and increased death. This is why influenza and pneumococcal vaccines have been introduced in every therapeutic algorithm and are recommended by international guidelines (110). Of the 18860 individuals exposed to the BNT162b2 vaccine (4), 1478 (7.8%) had chronic respiratory diseases, but, at the moment of the writing, no subgroup analysis has been published. So far, there should be no concern on the possible interaction between COPD pharmacological treatment and the approved vaccines. Therefore, the approved mRNA COVID-19 vaccines should be considered safe for patients with COPD.

The risk of SARS-CoV-2 infection in patients with asthma is still a matter of discussion (111, 112). Poorer outcomes in hospitalized patients with COVID-19 pneumonia and concomitant asthma were not related to the disease itself, but rather to older age and comorbidities, although corticosteroid therapy at high doses might be a confounding factor (113, 114). Biologics may be protective both against the SARS-CoV-2 infection, by down-regulating the angiotensin converting enzyme (ACE)2 (115) and the SARS-CoV-2-induced inflammatory response (116). Indeed, a low prevalence of COVID-19 was observed in European severe asthma cohorts (117, 118), although a recent report suggests that severe asthmatics receiving biologic treatments may suffer from a more severe disease compared with the general population (119). Current international guidelines suggest that all patients with asthma should be considered eligible for the approved mRNA COVID-19 vaccines (120). We highlight the need for individual highly specialized evaluation in patients with severe asthma, multiple comorbidities and long term treatment with systemic corticosteroids. Mild and moderate atopic patients (up to 30% of asthma patients) should not be excluded from the COVID-19 vaccination programs (121) due to the negligible risk of severe adverse reactions. However, allergic asthmatic patients with known hypersensitivity to multiple irritants or drugs, or that had past anaphylactic reactions to any injective treatment, should be thoroughly assessed for the risk/benefit ratio before receiving the vaccine (121).

Literature on SARS-CoV-2 infection susceptibility in patients with bronchiectasis is lacking. Nevertheless, compared with the general population, patients with bronchiectasis are at higher risk of developing viral, fungal and bacterial infections, and vaccination with mRNA vaccines should be considered safe and should be recommended. However, in patients with bronchiectasis secondary to immune defects or undergoing immune-suppressive treatments, a multi-disciplinary evaluation is suggested.

Last, sarcoidosis patients with a moderate/severe lung function impairment may have a heightened risk of SARS-CoV-2 infection (122). Small observational studies have demonstrated that anti-TNFα drugs are not related to worse outcomes or life threatening complications in sarcoidosis patients with COVID-19 (104, 123).

### Infectiologist’s Opinion

Vaccination is of course contraindicated in subjects on SARS-COV-2 acute infection. Controversy among scientists is ongoing regarding the opportunity to vaccinate subjects who already developed COVID-19 and recovered. Natural infection is able to elicit in most cases neutralizing antibodies (124). Moreover, previous infection may determine strong memory B cell responses despite low antibody titers in the blood and confer protection (125). In addition, it is still unclear what is the protective role played by cell mediated immunity but it may represent an efficient response to avoid re-infection (126).

Despite some vaccines have been approved and more are in development, no current clinical trial of a COVID-19 vaccine has enrolled immunocompromised patients. In immunosuppressed subjects vaccine efficacy is uncertain and immunity may be inappropriate even if safety of all other non-live vaccines available has been widely demonstrated (127, 128). Hosts lacking functional adaptive immune cells may be unable to generate a fully protective immune response to SARS-CoV-2 vaccines approved for use in the general population. In particular, anti-CD20 medications strongly affect the response to vaccines and adequate timing between drug administration and vaccination should be adopted (129, 130). For many other drugs, information on the response to vaccines is not available in literature. A further consideration is required for patients who have recently received high steroid dose therapy. In this case, the response to vaccines may be highly impaired and guidelines suggest waiting 4 weeks for inactivated vaccines and 3 months for live vaccines after steroids to perform vaccinations (131). We counsel to monitor antibody response about 1 month after the second dose of the SARS-COV-2 vaccine to evaluate immunogenicity. A potential approach in case of deficient response could be to add a third dose of vaccine possibly deferred at an optimal time after immunosuppressant delivery.

Durability of antibody response to SARS-COV-2 vaccination is currently uncertain in the general population and data are required to evaluate the duration of protection. It is likely that certain immunosuppressant drugs may reduce the duration of protection, therefore monitoring antibody titers even in the presence of immune response to vaccination should be considered to discern waning of immunity and possibly consider a booster dose of vaccine. Therefore, clinical trials are urgently needed to evaluate safety and immunogenicity of SARS-COV-2 vaccines with all the different immunosuppressive drugs used in immune-mediated diseases as each drug certainly affects immune response to vaccines to different degrees. Two important issues are to counsel patients to continue keeping the protective measures even after vaccination and to grant vaccination to all the people living with immunosuppressed subjects.

In summary, in our opinion, the risks and benefits of SARS-CoV-2 vaccines in immunocompromised patients should be weighed on a case-by-case basis, considering the incidence of the infection in the community, the level of immunosuppression of the patient, the underlying reason for immunosuppressive treatment and, last but not least, the approved vaccine formulation available at the moment of vaccination. Of course, any recommendations may change, based on the results of the approved vaccine trials.

## Conclusions

This opinion paper resulted from the need of our IMIDs Study Group to define an efficient strategy for the administration of the available COVID-19 vaccines based on what is already known about vaccinations for patients with IMIDs.

While the COVID-19 pandemic is still ongoing, bringing an explosion of high rates of hospitalization and mortality in many countries of the world, three COVID-19 vaccines have become available and have given us a glimpse of light and real hope to be able to effectively control virus diffusion. Therefore, it is not surprising that patients with IMIDs are asking us a number of questions about both the efficacy and safety of vaccines. The relative answers, therefore, require us to rapidly and promptly update our knowledge about the vaccines that are already available and those that are expected to become available in the coming months, in order to provide patients with clear and unambiguous answers. We are able to reassure our patients that, on the basis of the data currently available, SARS-CoV-2 vaccines seem safe, as demonstrated by the several millions of people that have already been vaccinated around the world since the first vaccine became available.

**Figure 1:**
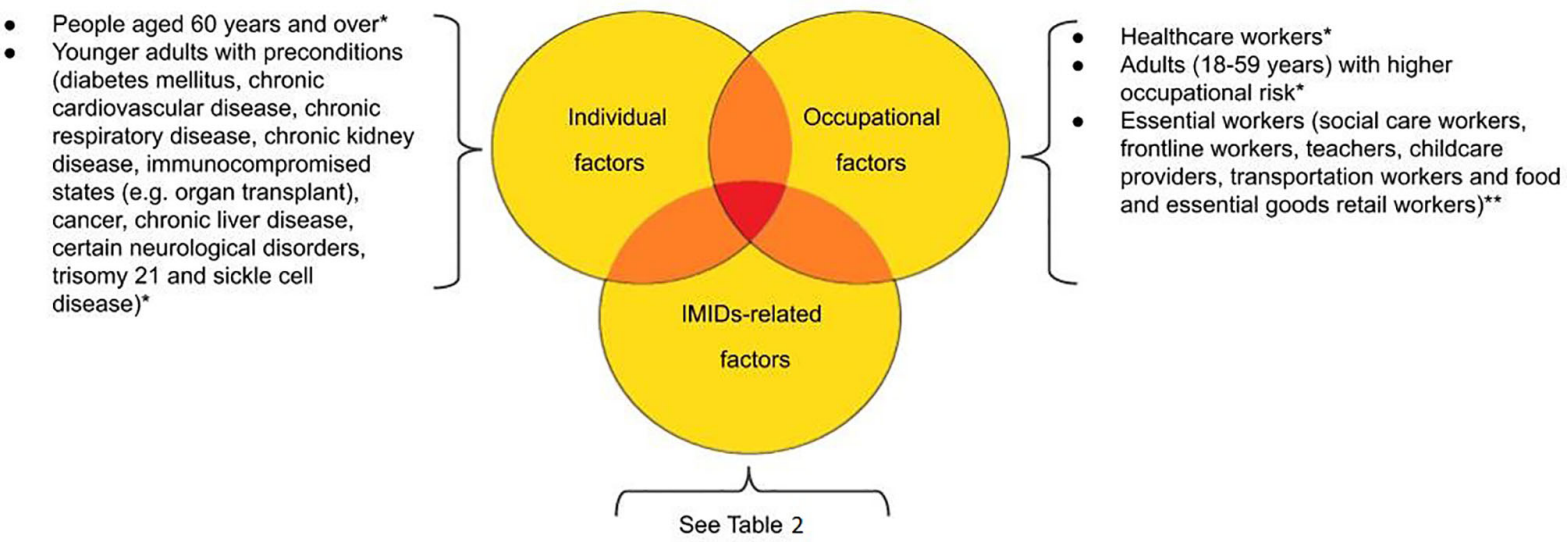
Prioritization proposal according to patients’ individual-, occupational- and IMIDs-related risk factors. *According to ECDC (European Centre for Disease Prevention and Control) Technical Report (https://www.ecdc.europa.eu/sites/default/files/documents/COVID-19-vaccination-and-prioritisation-strategies.pdf). **to evaluate; not included in the mathematical models.

Therefore, priority access to COVID-19 vaccine should be based on risk factors related to individual patients, different IMIDs, geographical differences, different occupational exposures, any factors that lead to the exclusion or delay of vaccine administration (e.g. patients with history of severe allergy, ongoing SARS-CoV-2 infection, patients on steroid or rituximab therapy). In general, patients with IMIDs can reasonably be encouraged to get vaccinated (**Figure 1**).

In addition to the general stratification, we propose a grading priority scale for patients in different disease situation, such as activity of the disease and medications (**Table 2**) and the timing of the vaccine based on the medications taken by the patient (**Table 1**). The speed with which COVID-19 vaccines are becoming available does not allow us to answer various questions such as:

- Are there any differences in the response to available vaccines, also related to different IMIDs?
- Can the different immunosuppressive and biological drugs used to treat IMIDs affect the nature and durability of the immune response after vaccination?
- In this patient setting, are two doses of vaccine sufficient, especially in those being treated with immunosuppressants and biologics?
- Can vaccines influence the severity of COVID-19?
- Considering that a high proportion of individuals infected with COVID-19 are asymptomatic, can vaccination also protect against asymptomatic infection?

**Table 2:** Suggested prioritization grading according to IMIDs-related risk factors.

IMIDs	IMIDs-related risk factors	Grading
**IBD**	IBD patients on biologic therapy (gut-selective) or immunosuppressants (azathioprine) or active IBD IBD patients on biologic therapy (non-gut-selective) Non-immunosuppressed inactive IBD patients (untreated or mesalamine-treated) Recent high-dose steroid therapy	+++ ++ + +/- (consider delay)
**Rheumatic Diseases**	Rheumatic disease patients on biologic therapy or immunosuppressants (including ≥ 10 mg/day of prednisone) Rheumatic disease patients with a history of severe infections Active rheumatic disease (e.g. high anti-CCP titer) Non-immunosuppressed patients with inactive disease Therapy with Rituximab	+++ ++ ++ + +/- (consider delay)
**Immune-mediated Glomerular Diseases**	IMGD patients on immunosuppressants (Cyclophosphamide, Mycophenolic acid, Cyclosporin/Tacrolimus) or active glomerular disease Stable, inactive IMGD patients (untreated) IMGD patients on anti-CD20 therapy (Rituximab) or recent high-dose steroid therapy	+++ + + (consider delay)
**Cutaneous Diseases**	Patients with autoimmune bullous diseases candidate to anti-CD20 therapy (vaccination should be scheduled at least 4 weeks before anti-CD20 therapy initiation) Patients on biologic therapy (anti-TNFα, anti-IL-17, anti-IL12/23) or conventional immunosuppressants (azathioprine, mycophenolate mofetil, cyclosporine) Patients with immune-mediated skin diseases treated with low dosages of systemic corticosteroids or untreated Patients treated with atopic dermatitis treated with dupilumab Patients with immune-mediated skin diseases treated with high dosages of systemic corticosteroids Patients with autoimmune bullous diseases treated with anti-CD20 therapy in the last 12-20 weeks	+++ ++ + + +/- (consider delay) +/- (consider delay)
**Ocular Diseases**	Patients with ocular conditions candidate to anti-CD20 therapy (vaccination should be scheduled at least 4 weeks before anti-CD20 therapy initiation) Patients with uveitis on biologic therapy (anti-TNFα, anti-IL-17, anti-IL12/23) or conventional immunosuppressants (azathioprine, mycophenolate mofetil, cyclosporine) Patients with uveitis with low dosages of systemic corticosteroids or untreated Patients with retinal diseases who need frequent access to the hospital for intravitreal treatment/monitoring Patients with acute autoimmune uveitis treated with high dosages of systemic corticosteroids Patients with autoimmune uveitis treated with anti-CD20 therapy in the last 12-20 weeks	+++ ++ + + +/- (consider delay) +/- (consider delay)
**Type 1 Diabetes Mellitus**	Adult patients with type 1 Diabetes Mellitus with poor glycemic control and/or cardiovascular or renal complications Adult patients with type 1 Diabetes Mellitus	+++ ++
**Chronic Pulmonary Diseases**	Severe COPD/Severe asthma/ILDs/IPF Mild-moderate COPD/asthma/bronchiectasis Bronchiectasis secondary to innate or acquired immunodeficiencies/ILDs with immunosuppressive treatments	+++ ++ +/- (multidisciplinary discussion/consider avoiding inactivated vaccine)

+++ indicates high priority patients.

++ indicates moderate priority patients.

+ indicates mild priority patients.

+/- consider delay and/or multidisciplinary management.

IMIDs, immune-mediated inflammatory diseases; IBD, inflammatory bowel disease; anti-CCP, anti–cyclic citrullinated peptide; IMGD, immune-mediated glomerular disease; TNFα, tumor necrosis factor; COPD, chronic obstructive pulmonary disease; ILDs, interstitial lung diseases; IPF, idiopathic pulmonary fibrosis.

To answer these questions, specific studies on different populations of patients with IMIDs are necessary. For this purpose, a specific Registry for SARS-CoV-2 vaccination prospectively enrolling all patients with IMIDs is crucial in order to obtain post-marketing surveillance of vaccine efficacy and safety.

## Collaborative Authors

The IMIDs Study Group includes Sandro Ardizzone, Rosanna Cannatelli, Stefania Carmagnola, Sabrina Caruso, Sara Castiglioni, Emilio Clementi, Piergiorgio Danelli, Dario Dilillo, Francesca Ferretti, Paolo Fiorina, Alice Frontali, Massimo Galli Maurizio Gallieni, Giovanni Genovese, Valeria Giorgi, Alessandro Invernizzi, Cristian Loretelli, Giovanni Maconi, Jeanette A. Maier, Angelo V. Marzano, Paola S. Morpurgo, Manuela Nebuloni, Dejan Radovanovic, Agostino Riva, Giuliano Rizzardini, Gianmarco Sabiu, Pierachille Santus, Pier Carlo Sarzi-Puttini, Giovanni Staurenghi, Michela Tedesco, Gianvincenzo Zuccotti.

## Data Availability Statement

The original contributions presented in the study are included in the article/supplementary material. Further inquiries can be directed to the corresponding author.

## Author Contributions

SA, PS-P, FF, RC, JM, and GM: planning the study, drafting the article, interpretation of data. All other authors: data collections, critical revision of the article for important intellectual content. All authors contributed to the article and approved the submitted version.

## Conflict of Interest

The authors declare that the research was conducted in the absence of any commercial or financial relationships that could be construed as a potential conflict of interest.

## References

[1] Fda. Pfizer-BioNTech COVID-19 VaccineEmergency Use Authorization Review Memorandum. (2020). Available at: https://www.fda.gov/media/144416/download.

[2] Fda. Moderna COVID-19 VaccineEmergency Use Authorization Review Memorandum. (2020). Available at: https://fda.report/media/144673/Moderna+COVID-19+Vaccine+review+memo.pdf.

[3] EMA. https://www.ema.europa.eu/en/medicines/human/EPAR/covid-19-vaccine-astrazeneca.

[4] Polack FP Thomas SJ Kitchin N Absalon J Gurtman A Lockhart S. Safety and Efficacy of the BNT162b2 mRNA Covid-19 Vaccine. N Engl J Med (2020) 383(27):2603–15. 10.1056/NEJMoa2034577 PMC7745181 33301246

[5] Baden LR El Sahly HM Essink B Kotloff K Frey S Novak R. Efficacy and Safety of the mRNA-1273 SARS-CoV-2 Vaccine. N Engl J Med (2020) 384(5):403–16. 10.1056/NEJMoa2035389 PMC7787219 33378609

[6] Jackson LA Anderson EJ Rouphael NG Roberts PC Makhene M Coler RN. An mRNA Vaccine against SARS-CoV-2 - Preliminary Report. N Engl J Med (2020) 383(20):1920–31. 10.1056/NEJMoa2022483 PMC7377258 32663912

[7] Voysey M Costa Clemens SA Madhi SA Weckx LY Folegatti PM Aley PK. Single-dose administration and the influence of the timing of the booster dose on immunogenicity and efficacy of ChAdOx1 nCoV-19 (AZD1222) vaccine: a pooled analysis of four randomised trials. Lancet. (2021) 397(10277):881–91. 10.1016/S0140-6736(21)00432-3 PMC7894131 33617777

[8] Fda. Fact sheet for healthcare providers administering vaccine (vaccination providers). (2020). Available at: https://www.fda.gov/media/144413/download.

[9] Castells MC Phillips EJ. Maintaining Safety with SARS-CoV-2 Vaccines. N Engl J Med (2020) 384(7):643–49. 10.1056/NEJMra2035343 PMC7787218 33378605

[10] Cdc. Interim Clinical Considerations for Use of mRNA COVID-19 Vaccines Currently Authorized in the United States. (2021). Available at: https://www.cdc.gov/vaccines/covid-19/downloads/summary-interim-clinical-considerations.pdf.

[11] Ema. Union Register of medicinal products for human use. (2020).

[12] Ema. EMA recommends COVID-19 Vaccine Moderna for authorisation in the EU. (2021). Available at: https://www.ema.europa.eu/en/news/ema-recommends-covid-19-vaccine-moderna-authorisation-eu#:~:text=EMA%20has%20recommended%20granting%20a,EMA%20has%20recommended%20for%20authorisation.

[13] https://www.rheumatology.org/Portals/0/Files/COVID-19-Vaccine-Clinical-Guidance-Rheumatic-Diseases-Summary.pdf. [Anonymous].

[14] Alexander JL Moran GW Gaya DR Raine T Hart A Kennedy NA. SARS-CoV-2 vaccination for patients with inflammatory bowel disease: a British Society of Gastroenterology Inflammatory Bowel Disease section and IBD Clinical Research Group position statement. Lancet Gastroenterol Hepatol (2021) 6(3):218–24. 10.1016/S2468-1253(21)00024-8 PMC7834976 33508241

[15] Waldman RA Creed M Sharp K Adalsteinnson J Imitola J Durso T. Letter in Reply: Toward a COVID-19 Vaccine Strategy for Pemphigus Patients on Rituximab. J Am Acad Dermatol (2020) 84(4):e197–8. 10.1016/j.jaad.2020.10.075 PMC7598732 33130180

[16] Santus P Saad M Damiani G Patella V Radovanovic D. Current and future targeted therapies for severe asthma: Managing treatment with biologics based on phenotypes and biomarkers. Pharmacol Res (2019) 146:104296–6. 10.1016/j.phrs.2019.104296 31173886

[17] Magro F Gionchetti P Eliakim R Ardizzone S Armuzzi A Barreiro-de Acosta M. Third European Evidence-based Consensus on Diagnosis and Management of Ulcerative Colitis. Part 1: Definitions, Diagnosis, Extra-intestinal Manifestations, Pregnancy, Cancer Surveillance, Surgery, and Ileo-anal Pouch Disorders. J Crohns Colitis (2017) 11(6):649–70. 10.1093/ecco-jcc/jjx008 28158501

[18] Ananthakrishnan AN McGinley EL. Infection-related hospitalizations are associated with increased mortality in patients with inflammatory bowel diseases. J Crohn’s Colitis (2013) 7:107–12. 10.1016/j.crohns.2012.02.015 22440891

[19] Rahier JF Magro F Abreu C Armuzzi A Ben-Horin S Chowers Y. Second European evidence-based consensus on the prevention, diagnosis and management of opportunistic infections in inflammatory bowel disease. J Crohns Colitis (2014) 8(6):443–68. 10.1016/j.crohns.2013.12.013 24613021

[20] Bonovas S Fiorino G Allocca M Lytras T Nikolopoulos GK Peyrin-Biroulet L. Biologic Therapies and Risk of Infection and Malignancy in Patients With Inflammatory Bowel Disease: A Systematic Review and Network Meta-analysis. Clin Gastroenterol Hepatol (2016) 14(10):1385–1397.e1310. 10.1016/j.cgh.2016.04.039 27189910

[21] Farraye FA Melmed GY Lichtenstein GR Kane SV. ACG Clinical Guideline: Preventive Care in Inflammatory Bowel Disease. Am J Gastroenterol (2017) 112(2):241–58. 10.1038/ajg.2016.537 28071656

[22] Maconi G Bosetti C De Monti A Boyapati RK Shelton E Piazza N. Risk of COVID 19 in patients with inflammatory bowel diseases compared to a control population. Dig Liver Dis (2020) 53(3):263–70. 10.1016/j.dld.2020.12.013 PMC7762705 33483259

[23] Bezzio C Saibeni S Variola A Allocca M Massari A Gerardi V. Outcomes of COVID-19 in 79 patients with IBD in Italy: an IG-IBD study. Gut (2020) 69(7):1213–7. 10.1136/gutjnl-2020-321411 32354990

[24] Neurath MF. COVID-19 and immunomodulation in IBD. Gut (2020) 69:1335–42. 10.1136/gutjnl-2020-321269 PMC7211083 32303609

[25] Ungaro RC Brenner EJ Gearry RB Kaplan GG Kissous-Hunt M Lewis JD. Effect of IBD medications on COVID-19 outcomes: results from an international registry. Gut (2020) 70(4):725–32. 10.1136/gutjnl-2020-322539 PMC8136807 33082265

[26] Lichtenstein L Ron Y Kivity S Ben-Horin S Israeli E Fraser GM. Infliximab-Related Infusion Reactions: Systematic Review. J Crohns Colitis (2015) 9(9):806–15. 10.1093/ecco-jcc/jjv096 PMC4558633 26092578

[27] Melmed GY Agarwal N Frenck RW Ippoliti AF Ibanez P Papadakis KA. Immunosuppression impairs response to pneumococcal polysaccharide vaccination in patients with inflammatory bowel disease. Am J Gastroenterol (2010) 105(1):148–54. 10.1038/ajg.2009.523 19755964

[28] Cullen G Bader C Korzenik JR Sands BE. Serological response to the 2009 H1N1 influenza vaccination in patients with inflammatory bowel disease. Gut (2012) 61(3):385–91. 10.1136/gutjnl-2011-300256 21757451

[29] Cleveland NK Rodriquez D Wichman A Pan I Melmed GY Rubin DT. Many Inflammatory Bowel Disease Patients Are Not Immune to Measles or Pertussis. Dig Dis Sci (2016) 61(10):2972–6. 10.1007/s10620-016-4275-2 27557706

[30] Melmed GY. Vaccinations while on thiopurines: some protection is better than none. Am J Gastroenterol (2012) 107:141–2. 10.1038/ajg.2011.320 22218037

[31] Melmed GY Rubin DT McGovern DPB. Winter Is Coming! Clinical, Immunologic, and Practical Considerations for Vaccinating Patients With Inflammatory Bowel Disease During the Coronavirus Disease-2019 Pandemic. Gastroenterology (2020) 160(3):639–44. 10.1053/j.gastro.2020.10.013 PMC7553998 33065064

[32] Kumar A Quraishi MN Segal JP Raine T Brookes MJ. COVID-19 vaccinations in patients with inflammatory bowel disease. Lancet Gastroenterol Hepatol (2020) 5(11):965–6. 10.1016/S2468-1253(20)30295-8 PMC7505629 32971020

[33] Sarzi-Puttini P Marotto D Antivalle M Salaffi F Atzeni F Maconi G. How to handle patients with autoimmune rheumatic and inflammatory bowel diseases in the COVID-19 era: An expert opinion. Autoimmun Rev (2020) 19(7):102574–4. 10.1016/j.autrev.2020.102574 PMC7200131 32376399

[34] Walsh EE Frenck RW Jr Falsey AR Kitchin N Absalon J Gurtman A. Safety and Immunogenicity of Two RNA-Based Covid-19 Vaccine Candidates. N Engl J Med (2020) 383(25):2439–50. 10.1056/NEJMoa2027906 PMC7583697 33053279

[35] Sarzi-Puttini P Marotto D Caporali R Montecucco CM Favalli EG Franceschini F. Prevalence of COVID infections in a population of rheumatic patients from Lombardy and Marche treated with biological drugs or small molecules: A multicentre retrospective study. J Autoimmun (2021) 116:102545–5. 10.1016/j.jaut.2020.102545 PMC7506330 32972804

[36] Gianfrancesco M Hyrich KL Al-Adely S Carmona L Danila MI Gossec L. Characteristics associated with hospitalisation for COVID-19 in people with rheumatic disease: data from the COVID-19 Global Rheumatology Alliance physician-reported registry. Ann Rheum Dis (2020) 79(7):859–66. 10.1136/annrheumdis-2020-217871 PMC7299648 32471903

[37] Akiyama S Hamdeh S Micic D Sakuraba A. Prevalence and clinical outcomes of COVID-19 in patients with autoimmune diseases: a systematic review and meta-analysis. Ann Rheum Dis (2020) 13:annrheumdis-2020-218946. 10.1136/annrheumdis-2020-218946 33051220

[38] Kapetanovic MC Saxne T Sjoholm A Truedsson L Jonsson G Geborek P. Influence of methotrexate, TNF blockers and prednisolone on antibody responses to pneumococcal polysaccharide vaccine in patients with rheumatoid arthritis. Rheumatol (Oxford) (2006) 45(1):106–11. 10.1093/rheumatology/kei193 16287919

[39] Kapetanovic MC Roseman C Jonsson G Truedsson L Saxne T Geborek P. Antibody response is reduced following vaccination with 7-valent conjugate pneumococcal vaccine in adult methotrexate-treated patients with established arthritis, but not those treated with tumor necrosis factor inhibitors. Arthritis Rheum (2011) 63(12):3723–32. 10.1002/art.30580 21834061

[40] Alten R Bingham 3CO Cohen SB Curtis JR Kelly S Wong D. Antibody response to pneumococcal and influenza vaccination in patients with rheumatoid arthritis receiving abatacept. BMC Musculoskelet Disord (2016) 17:231–1. 10.1186/s12891-016-1082-z PMC4880815 27229685

[41] Migita K Akeda Y Akazawa M Tohma S Hirano F Ideguchi H. Effect of abatacept on the immunogenicity of 23-valent pneumococcal polysaccharide vaccination (PPSV23) in rheumatoid arthritis patients. Arthritis Res Ther (2015) 17:357–7. 10.1186/s13075-015-0863-3 PMC4675027 26653668

[42] Mori S Ueki Y Hirakata N Oribe M Hidaka T Oishi K. Impact of tocilizumab therapy on antibody response to influenza vaccine in patients with rheumatoid arthritis. Ann Rheum Dis (2012) 71(12):2006–10. 10.1136/annrheumdis-2012-201950 PMC3595981 22887851

[43] Subesinghe S Bechman K Rutherford AI Goldblatt D Galloway JB. A Systematic Review and Metaanalysis of Antirheumatic Drugs and Vaccine Immunogenicity in Rheumatoid Arthritis. J Rheumatol (2018) 45(6):733–44. 10.3899/jrheum.170710 29545454

[44] Iwamoto M Homma S Onishi S Kamata Y Nagatani K Yamagata Z. Low level of seroconversion after a novel influenza A/H1N1/2009 vaccination in Japanese patients with rheumatoid arthritis in the 2009 season. Rheumatol Int (2012) 32(11):3691–4. 10.1007/s00296-011-2118-1 21881985

[45] Franca IL Ribeiro AC Aikawa NE Saad CG Moraes JC Goldstein-Schainberg C. TNF blockers show distinct patterns of immune response to the pandemic influenza A H1N1 vaccine in inflammatory arthritis patients. Rheumatol (Oxford) (2012) 51(11):2091–8. 10.1093/rheumatology/kes202 PMC7313849 22908326

[46] Ribeiro AC Guedes LK Moraes JC Saad CG Aikawa NE Calich AL. Reduced seroprotection after pandemic H1N1 influenza adjuvant-free vaccination in patients with rheumatoid arthritis: implications for clinical practice. Ann Rheum Dis (2011) 70(12):2144–7. 10.1136/ard.2011.152983 21859696

[47] Ribeiro AC Laurindo IM Guedes LK Saad CG Moraes JC Silva CA. Abatacept and reduced immune response to pandemic 2009 influenza A/H1N1 vaccination in patients with rheumatoid arthritis. Arthritis Care Res (Hoboken) (2013) 65(3):476–80. 10.1002/acr.21838 22949223

[48] Bingham 3rd CO Rizzo W Kivitz A Hassanali A Upmanyu R Klearman M. Humoral immune response to vaccines in patients with rheumatoid arthritis treated with tocilizumab: results of a randomised controlled trial (VISARA). Ann Rheum Dis (2015) 74(5):818–22. 10.1136/annrheumdis-2013-204427 PMC4392200 24448345

[49] Friedman MA Winthrop K. Vaccinations for rheumatoid arthritis. Curr Opin Rheumatol (2016) 28:330–6. 10.1097/BOR.0000000000000281 26986246

[50] Benucci M Quartuccio L Li Gobbi F Damiani A Grossi V Infantino M. Persistence of rT-PCR-SARS-CoV-2 infection and delayed serological response, as a possible effect of rituximab according to the hypothesis of Schulze-Koops et al. Ann Rheum Dis (2020) 4:annrheumdis-2020-218590. 10.1136/annrheumdis-2020-218590 32753417

[51] Baker D Roberts CAK Pryce G Kang AS Marta M Reyes S. COVID-19 vaccine-readiness for anti-CD20-depleting therapy in autoimmune diseases. Clin Exp Immunol (2020) 202(2):149–61. 10.1111/cei.13495 PMC7405500 32671831

[52] Couser WG. Basic and translational concepts of immune-mediated glomerular diseases. J Am Soc Nephrol (2012) 23:381–99. 10.1681/ASN.2011030304 22282593

[53] Floege J Barbour SJ Cattran DC Hogan JJ Nachman PH Tang SCW. Management and treatment of glomerular diseases (part 1): conclusions from a Kidney Disease: Improving Global Outcomes (KDIGO) Controversies Conference. Kidney Int (2019) 95(2):268–80. 10.1016/j.kint.2018.10.018 30665568

[54] Mastalerz-Migas A Bujnowska-Fedak M Brydak LB. Immune efficacy of first and repeat trivalent influenza vaccine in healthy subjects and hemodialysis patients. Adv Exp Med Biol (2015) 836:47–54. 10.1007/5584_2014_36 25248348

[55] Carugno A Raponi F Locatelli AG Vezzoli P Gambini DM Di Mercurio M. No evidence of increased risk for Coronavirus Disease 2019 (COVID-19) in patients treated with Dupilumab for atopic dermatitis in a high-epidemic area - Bergamo, Lombardy, Italy. J Eur Acad Dermatol Venereol (2020) 34(9):e433–4. 10.1111/jdv.16552 PMC7267230 32339362

[56] Krueger KM Ison MG Ghossein C. Practical Guide to Vaccination in All Stages of CKD, Including Patients Treated by Dialysis or Kidney Transplantation. Am J Kidney Dis (2020) 75:417–25. 10.1053/j.ajkd.2019.06.014 31585683

[57] McDonald HI Thomas SL Nitsch D. Chronic kidney disease as a risk factor for acute community-acquired infections in high-income countries: a systematic review. BMJ Open (2014) 4:e004100. 10.1136/bmjopen-2013-004100 PMC3996818 24742975

[58] Dalrymple LS Mu Y Nguyen DV Romano PS Chertow GM Grimes B. Risk Factors for Infection-Related Hospitalization in In-Center Hemodialysis. Clin J Am Soc Nephrol (2015) 10:2170–80. 10.2215/CJN.03050315 PMC4670763 26567370

[59] Waldman M Soler MJ Garcia-Carro C Lightstone L Turner-Stokes T Griffith M. Results from the IRoc-GN international registry of patients with COVID-19 and glomerular disease suggest close monitoring. Kidney Int (2021) 99(1):227–37. 10.1016/j.kint.2020.10.032 PMC7833801 33181156

[60] Houot R Levy R Cartron G Armand P. Could anti-CD20 therapy jeopardise the efficacy of a SARS-CoV-2 vaccine? Eur J Cancer (2020) 136:4–6. 10.1016/j.ejca.2020.06.017 32619884 PMC7315961

[61] Cho A Bradley B Kauffman R Priyamvada L Kovalenkov Y Feldman R. Robust memory responses against influenza vaccination in pemphigus patients previously treated with rituximab. JCI Insight (2017) 2(12):e93222. 10.1172/jci.insight.93222 PMC5470882 28614800

[62] Torres T Puig L. Managing Cutaneous Immune-Mediated Diseases During the COVID-19 Pandemic. Am J Clin Dermatol (2020) 21:307–11. 10.1007/s40257-020-00514-2 PMC7147535 32277351

[63] Cho SI Kim YE Jo SJ. Association of COVID-19 with skin diseases and relevant biologics: a cross-sectional study using nationwide claim data in South Korea. Br J Dermatol (2020) 184(2):296–303. 10.1111/bjd.19507 32875557 PMC9213995

[64] Thyssen JP Vestergaard C Barbarot S de Bruin-Weller MS Bieber T Taieb A. European Task Force on Atopic Dermatitis (ETFAD): position on vaccination of adult patients with atopic dermatitis against COVID-19 (SARS-CoV-2) being treated with systemic medication and biologics. J Eur Acad Dermatol Venereol (2021) 15:10.1111/jdv.17167. 10.1111/jdv.17167 PMC8014632 33587756

[65] Seltzer JA Okeke CAV Perry JD Shipman WD Okoye GA Byrd AS. Exploring the risk of severe COVID-19 infection in patients with hidradenitis suppurativa. J Am Acad Dermatol (2020) 83(2):e153–4. 10.1016/j.jaad.2020.05.012 PMC7205672 32389715

[66] Veenstra J Buechler CR Robinson G Chapman S Adelman M Tisack A. Antecedent immunosuppressive therapy for immune-mediated inflammatory diseases in the setting of a COVID-19 outbreak. J Am Acad Dermatol (2020) 83(6):1696–703. 10.1016/j.jaad.2020.07.089 PMC7385924 32735965

[67] Gisondi P Piaserico S Naldi L Dapavo P Conti A Malagoli P. Incidence rates of hospitalization and death from COVID-19 in patients with psoriasis receiving biological treatment: A Northern Italy experience. J Allergy Clin Immunol (2020) 147(2):558–60.e1. 10.1016/j.jaci.2020.10.032 PMC7644231 33160968

[68] Marzano AV Moltrasio C Genovese G Muratori S Dapavo P Fabbrocini G. Hidradenitis suppurativa and adalimumab in the COVID-19 era. Eur J Dermatol (2020) 30(6):748–9. 10.1684/ejd.2020.3927 PMC7880651 33337329

[69] Wang C Rademaker M Baker C Foley P. COVID-19 and the use of immunomodulatory and biologic agents for severe cutaneous disease: An Australian/New Zealand consensus statement. Australas J Dermatol (2020) 61(3):210–6. 10.1111/ajd.13313 PMC7262046 32255510

[70] Reynolds SD Mathur AN Chiu YE Brandling-Bennett HA Pope E Siegel MP. Systemic immunosuppressive therapy for inflammatory skin diseases in children: Expert consensus-based guidance for clinical decision-making during the COVID-19 pandemic. Pediatr Dermatol (2020) 37(3):424–34. 10.1111/pde.14202 32320494

[71] Kasperkiewicz M Schmidt E Fairley JA Joly P Payne AS Yale ML. Expert recommendations for the management of autoimmune bullous diseases during the COVID-19 pandemic. J Eur Acad Dermatol Venereol (2020) 34(7):e302–3. 10.1111/jdv.16525 PMC7267551 32333823

[72] Joly P Horvath B Patsatsi A Uzun S Bech R Beissert S. Updated S2K guidelines on the management of pemphigus vulgaris and foliaceus initiated by the european academy of dermatology and venereology (EADV). J Eur Acad Dermatol Venereol (2020) 34(9):1900–13. 10.1111/jdv.16752 32830877

[73] Wollenberg A Flohr C Simon D Cork MJ Thyssen JP Bieber T. European Task Force on Atopic Dermatitis statement on severe acute respiratory syndrome coronavirus 2 (SARS-Cov-2) infection and atopic dermatitis. J Eur Acad Dermatol Venereol (2020) 34(6):e241–2. 10.1111/jdv.16411 32223003

[74] Friedman MA Winthrop KL. Vaccines and Disease-Modifying Antirheumatic Drugs: Practical Implications for the Rheumatologist. Rheum Dis Clin North Am (2017) 43:1–13. 10.1016/j.rdc.2016.09.003 27890167

[75] Gunes AT Fetil E Akarsu S Ozbagcivan O Babayeva L. Possible Triggering Effect of Influenza Vaccination on Psoriasis. J Immunol Res (2015) 2015:258430–0. 10.1155/2015/258430 PMC4562095 26380315

[76] Sbidian E Eftekahri P Viguier M Laroche L Chosidow O Gosselin P. National survey of psoriasis flares after 2009 monovalent H1N1/seasonal vaccines. Dermatology (2014) 229(2):130–5. 10.1159/000362808 25171322

[77] Marzano AV Cassano N Genovese G Moltrasio C Vena GA. Cutaneous manifestations in patients with COVID-19: a preliminary review of an emerging issue. Br J Dermatol (2020) 183(3):431–42. 10.1111/bjd.19264 PMC7300648 32479680

[78] Genovese G Moltrasio C Berti E Marzano AV. Skin Manifestations Associated with COVID-19: Current Knowledge and Future Perspectives. Dermatology (2021) 237(1):1–12. 10.1159/000512932 33232965 PMC7801998

[79] Dick AD Rosenbaum JT Al-Dhibi HA Belfort R Brézin AP Chee SP. Guidance on Noncorticosteroid Systemic Immunomodulatory Therapy in Noninfectious Uveitis: Fundamentals Of Care for UveitiS (FOCUS) Initiative. Ophthalmology (2018) 125(5):757–73. 10.1016/j.ophtha.2017.11.017 29310963

[80] Zierhut M De Smet MD Gupta V Pavesio C Nguyen QD Chee SP. Evolving Consensus Experience of the IUSG-IOIS-FOIS with Uveitis in the Time of COVID-19 Infection. Ocul Immunol Inflammation (2020) 28(5):709–13. 10.1080/09273948.2020.1780273 32721206

[81] Casagrande M Fitzek A Puschel K Aleshcheva G Schultheiss HP Berneking L. Detection of SARS-CoV-2 in Human Retinal Biopsies of Deceased COVID-19 Patients. Ocul Immunol Inflammation (2020) 28(5):721–5. 10.1080/09273948.2020.1770301 32469258

[82] Invernizzi A Torre A Parrulli S Zicarelli F Schiuma M Colombo V. Retinal findings in patients with COVID-19: Results from the SERPICO-19 study. EClinicalMedicine (2020) 27:100550–0. 10.1016/j.eclinm.2020.100550 PMC7502280 32984785

[83] Lani-Louzada R Ramos C Cordeiro RM Sadun AA. Retinal changes in COVID-19 hospitalized cases. PloS One (2020) 15(12):e0243346–e0243346. 10.1371/journal.pone.0243346 33270751 PMC7714146

[84] Invernizzi A Pellegrini M Messenio D Cereda M Olivieri P Brambilla AM. Impending Central Retinal Vein Occlusion in a Patient with Coronavirus Disease 2019 (COVID-19). Ocul Immunol Inflammation (2020) 28(8):1290–2. 10.1080/09273948.2020.1807023 32976055

[85] Gaba WH Ahmed D Al Nuaimi RK Dhanhani AA Eatamadi H. Bilateral Central Retinal Vein Occlusion in a 40-Year-Old Man with Severe Coronavirus Disease 2019 (COVID-19) Pneumonia. Am J Case Rep (2020) 21:e927691–e927691. 10.12659/AJCR.927691 33116072 PMC7603800

[86] Viola F Milella P Giuffrida FP Ganci S Invernizzi A. The impact of coronavirus disease (COVID-19) pandemic on intravitreal injections treatment for macular diseases: report from a referral hospital in Milan. Retina (2020) 41(4):701–5. 10.1097/IAE.0000000000002941 32796445

[87] Romano F Monteduro D Airaldi M Zicarelli F Parrulli S Cozzi M. Increased Number of Submacular Hemorrhages as a Consequence of Coronavirus Disease 2019 Lockdown. Ophthalmol Retina (2020) 4(12):1209–10. 10.1016/j.oret.2020.06.027 PMC7315987 32593777

[88] Jackson J Silvestri G Stevenson M Sinton J Witherow J McCann R. COVID-19: The regional impact of COVID-19 on the certification of vision impairment in Northern Ireland. Ophthalmic Physiol Opt (2021) 41(1):136–43. 10.1111/opo.12757 33165967

[89] Gery I Caspi RR. Tolerance Induction in Relation to the Eye. Front Immunol (2018) 9:2304. 10.3389/fimmu.2018.02304 30356688 PMC6189330

[90] Cunningham ET Jr Moorthy RS. Vaccine-Associated Posterior Uveitis. Retina (2020) 40:595–8. 10.1097/IAE.0000000000002816 32221173

[91] Izzi-Engbeaya C Distaso W Amin A Yang W Idowu O Kenkre JS. Adverse outcomes in COVID-19 and diabetes: a retrospective cohort study from three London teaching hospitals. BMJ Open Diabetes Res Care (2021) 9(1):e001858. 10.1136/bmjdrc-2020-001858 PMC7789097 33408084

[92] Carey IM Critchley JA DeWilde S Harris T Hosking FJ Cook DG. Risk of Infection in Type 1 and Type 2 Diabetes Compared With the General Population: A Matched Cohort Study. Diabetes Care (2018) 41(3):513–21. 10.2337/dc17-2131 29330152

[93] Critchley JA Carey IM Harris T DeWilde S Hosking FJ Cook DG. Glycemic Control and Risk of Infections Among People With Type 1 or Type 2 Diabetes in a Large Primary Care Cohort Study. Diabetes Care (2018) 41(10):2127–35. 10.2337/dc18-0287 30104296

[94] Casqueiro J Alves C. Infections in patients with diabetes mellitus: A review of pathogenesis. Indian J Endocrinol Metab (2012) 16 Suppl 1:S27–36. 10.4103/2230-8210.94253 PMC3354930 22701840

[95] Standards of Medical Care in Diabetes—2020 Abridged for Primary Care Providers American Diabetes Association. Clin Diabetes (2020) 38(1):10–38. 10.2337/cd20-as01 31975748 PMC6969656

[96] Sid. Standard Italiani per la Cura del Diabete Mellito - 2018. (2018). Available at: https://aemmedi.it/wp-content/uploads/2009/06/AMD-Standard-unico1.pdf.

[97] Eibl N Spatz M Fischer GF Mayr WR Samstag A Wolf HM. Impaired primary immune response in type-1 diabetes: results from a controlled vaccination study. Clin Immunol (2002) 103(3 Pt 1):249–59. 10.1006/clim.2002.5220 12173299

[98] el-Madhun AS Cox RJ Seime A Sovik O Haaheim LR. Systemic and local immune responses after parenteral influenza vaccination in juvenile diabetic patients and healthy controls: results from a pilot study. Vaccine (1998) 16(2-3):156–60. 10.1016/s0264-410x(97)88328-4 9607024

[99] Remschmidt C Wichmann O Harder T. Vaccines for the prevention of seasonal influenza in patients with diabetes: systematic review and meta-analysis. BMC Med (2015) 13:53–3. 10.1186/s12916-015-0295-6 PMC4373029 25857236

[100] Cardona-Hernandez R Cherubini V Iafusco D Schiaffini R Luo X Maahs DM. Children and youth with diabetes are not at increased risk for hospitalization due to COVID-19. Pediatr Diabetes (2020) 22(2):202–6. 10.1111/pedi.13158 PMC7753354 33205546

[101] Barron E Bakhai C Kar P Weaver A Bradley D Ismail H. Associations of type 1 and type 2 diabetes with COVID-19-related mortality in England: a whole-population study. Lancet Diabetes Endocrinol (2020) 8(10):813–22. 10.1016/S2213-8587(20)30272-2 PMC7426088 32798472

[102] Holman N Knighton P Kar P O'Keefe J Curley M Weaver A. Risk factors for COVID-19-related mortality in people with type 1 and type 2 diabetes in England: a population-based cohort study. Lancet Diabetes Endocrinol (2020) 8(10):823–33. 10.1016/S2213-8587(20)30271-0 PMC7426091 32798471

[103] Solerte SB D’Addio F Trevisan R Lovati E Rossi A Pastore I. Sitagliptin Treatment at the Time of Hospitalization Was Associated With Reduced Mortality in Patients With Type 2 Diabetes and COVID-19: A Multicenter, Case-Control, Retrospective, Observational Study. Diabetes Care (2020) 43(12):2999–3006. 10.2337/dc20-1521 32994187 PMC7770266

[104] Southern BD. Patients with interstitial lung disease and pulmonary sarcoidosis are at high risk for severe illness related to COVID-19. Cleve Clin J Med (2020). 10.3949/ccjm.87a.ccc026 32409436

[105] Drake TM Docherty AB Harrison EM Quint JK Adamali H Agnew S. Outcome of Hospitalization for COVID-19 in Patients with Interstitial Lung Disease. An International Multicenter Study. Am J Respir Crit Care Med (2020) 202(12):1656–65. 10.1164/rccm.202007-2794OC PMC7737581 33007173

[106] Gallay L Uzunhan Y Borie R Lazor R Rigaud P Marchand-Adam S. Risk Factors for Mortality after COVID-19 in Patients with Preexisting Interstitial Lung Disease. Am J Respir Crit Care Med (2021) 203(2):245–9. 10.1164/rccm.202007-2638LE PMC7874431 33252997

[107] The European Idiopathic Pulmonary Fibrosis & Related Disorders Federation (EU-IPFF) and the European Reference Network on Rare Respiratory Diseases (ERN-Lung). Pulmonary fibrosis patients should be given priority in COVID-19 vaccination programmes: A Joint Statement. https://www.eu-ipff.org/news/article/pulmonary-fibrosis-patients-should-be-given-priority-in-covid-19-vaccination-programmes-a-joint-statement-1 [Accessed on April 2021].

[108] Hippisley-Cox J Young D Coupland C Channon KM Tan PS Harrison DA. Risk of severe COVID-19 disease with ACE inhibitors and angiotensin receptor blockers: cohort study including 8.3 million people. Heart (2020) 106(19):1503–11. 10.1136/heartjnl-2020-317393 PMC7509391 32737124

[109] Graziani D Soriano JB Del Rio-Bermudez C Morena D Diaz T Castillo M. Characteristics and Prognosis of COVID-19 in Patients with COPD. J Clin Med (2020) 9(10):3259. 10.3390/jcm9103259 PMC7600734 33053774

[110] Global Strategy for Prevention, diagnosis and management of chronic obstructive pulmonary disease (GOLD). (2021). Available at: https://goldcopd.org/wp-content/uploads/2020/11/GOLD-REPORT-2021-v1.1-25Nov20_WMV.pdf.

[111] Timberlake DT Strothman K Grayson MH. Asthma, severe acute respiratory syndrome coronavirus-2 and coronavirus disease 2019. Curr Opin Allergy Clin Immunol (2021) 21(2):182–7. 10.1097/ACI.0000000000000720 33399389

[112] Williamson EJ Walker AJ Bhaskaran K Bacon S Bates C Morton CE. Factors associated with COVID-19-related death using OpenSAFELY. Nature (2020) 584(7821):430–6. 10.1038/s41586-020-2521-4 PMC7611074 32640463

[113] Izquierdo JL Almonacid C Gonzalez Y Del Rio-Bermudez C Ancochea J Cardenas R. The Impact of COVID-19 on Patients with Asthma. Eur Respir J (2020) 57(3):2003142. 10.1183/13993003.03142-2020 PMC7651839 33154029

[114] Eger K Bel EH. Asthma and COVID-19: do we finally have answers? Eur Respir J (2020) 57(3):2004451. 10.1183/13993003.04451-2020 PMC7778875 33380511

[115] Jackson DJ Busse WW Bacharier LB Kattan M O'Connor GT Wood RA. Association of respiratory allergy, asthma, and expression of the SARS-CoV-2 receptor ACE2. J Allergy Clin Immunol (2020) 146(1):203–6.e203. 10.1016/j.jaci.2020.04.009 32333915 PMC7175851

[116] Carli G Cecchi L Stebbing J Parronchi P Farsi A. Is asthma protective against COVID-19? Allergy (2020) 76(3):866–8. 10.1111/all.14426 PMC7300712 32479648

[117] Heffler E Detoraki A Contoli M Papi A Paoletti G Malipiero G. COVID-19 in Severe Asthma Network in Italy (SANI) patients: Clinical features, impact of comorbidities and treatments. Allergy (2020) 76(3):887–92. 10.1111/all.14532 PMC7436509 32738147

[118] Hanon S Brusselle G Deschampheleire M Louis R Michils A Peche R. COVID-19 and biologics in severe asthma: data from the Belgian Severe Asthma Registry. Eur Respir J (2020) 56(6):2002857. 10.1183/13993003.02857-2020 PMC7712385 33093115

[119] Eger K Hashimoto S Braunstahl GJ Brinke AT Patberg KW Beukert A. Poor outcome of SARS-CoV-2 infection in patients with severe asthma on biologic therapy. Respir Med (2020) 177:106287–7. 10.1016/j.rmed.2020.106287 PMC7833566 33388603

[120] Global initiative for Asthma. Interim guidance about COVID-19 and Asthma. Updated December 20th 2020. (2020). Available at: https://ginasthma.org/wp-content/uploads/2020/12/GINA-interim-guidance-on-COVID-19-and-asthma-20_12_20.pdf.

[121] Klimek L Jutel M Akdis CA Bousquet J Akdis M Torres-Jaen M. ARIA-EAACI statement on severe allergic reactions to COVID-19 vaccines - an EAACI-ARIA position paper. Allergy (2020). 10.1111/all.14726 33378789

[122] Morgenthau AS Levin MA Freeman R Reich DL Klang E. Moderate or Severe Impairment in Pulmonary Function is Associated with Mortality in Sarcoidosis Patients Infected with SARSCoV2. Lung (2020) 198(5):771–5. 10.1007/s00408-020-00392-9 PMC7484928 32915271

[123] Manansala M Ascoli C Alburquerque AG Perkins D Mirsaedi M Finn P. Case Series: COVID-19 in African American Patients With Sarcoidosis. Front Med (Lausanne) (2020) 7:588527. 10.3389/fmed.2020.588527 33251236 PMC7672207

[124] Piccoli L Park YJ Tortorici MA Czudnochowski N Walls AC Beltramello M. Mapping Neutralizing and Immunodominant Sites on the SARS-CoV-2 Spike Receptor-Binding Domain by Structure-Guided High-Resolution Serology. Cell (2020) 183(4):1024–42.e1021. 10.1016/j.cell.2020.09.037 32991844 PMC7494283

[125] Nguyen-Contant P Embong AK Kanagaiah P Chaves FA Yang H Branche AR. S Protein-Reactive IgG and Memory B Cell Production after Human SARS-CoV-2 Infection Includes Broad Reactivity to the S2 Subunit. mBio (2020) 11(5):e01991–20. 10.1128/mBio.01991-20 PMC7520599 32978311

[126] Rodda LB Netland J Shehata L Pruner KB Morawski PA Thouvenel CD. Functional SARS-CoV-2-Specific Immune Memory Persists after Mild COVID-19. Cell (2021) 184(1):169–83.e117. 10.1016/j.cell.2020.11.029 33296701 PMC7682481

[127] Rondaan C Furer V Heijstek MW Agmon-Levin N Bijl M Breedveld FC. Efficacy, immunogenicity and safety of vaccination in adult patients with autoimmune inflammatory rheumatic diseases: a systematic literature review for the 2019 update of EULAR recommendations. RMD Open (2019) 5(2):e001035–e001035. 10.1136/rmdopen-2019-001035 31565247 PMC6744079

[128] Riva A Barcella V Benatti SV Capobianco M Capra R Cinque P. Vaccinations in patients with multiple sclerosis: A Delphi consensus statement. Mult Scler (2021) 27(3):347–59. 10.1177/1352458520952310 32940128

[129] Nazi I Kelton JG Larché M Snider DP Heddle NM Crowther MA. The effect of rituximab on vaccine responses in patients with immune thrombocytopenia. Blood (2013) 122(11):1946–53. 10.1182/blood-2013-04-494096 PMC3773242 23851398

[130] Bar-Or A Calkwood JC Chognot C Evershed J Fox EJ Herman A. Effect of ocrelizumab on vaccine responses in patients with multiple sclerosis: The VELOCE study. Neurology (2020) 95(14):e1999–2008. 10.1212/WNL.0000000000010380 PMC7843152 32727835

[131] Rubin LG Levin MJ Ljungman P Davies EG Avery R Tomblyn M. 2013 IDSA clinical practice guideline for vaccination of the immunocompromised host. Clin Infect Dis (2014) 58(3):309–18. 10.1093/cid/cit816 24421306

